# Clinical Outcomes of Hepatic Squamous Cell Carcinoma With Fibroblast Growth Factor Receptor 2 (FGFR2) Mutation: A Case Report

**DOI:** 10.7759/cureus.78024

**Published:** 2025-01-26

**Authors:** Ryogo Minami, Masamichi Kimura, Koji Nishikawa, Jun Imamura, Kiminori Kimura

**Affiliations:** 1 Hepatology, Tokyo Metropolitan Cancer and Infectious Diseases Center, Komagome Hospital, Tokyo, JPN

**Keywords:** erdafitinib, fgfr2, liver cancer, squamous cell carcinoma, tumor profiling

## Abstract

Primary squamous cell carcinoma of the liver (PSCCL) is an extremely rare disease with a poor prognosis. To date, few cancer-related genetic abnormalities in PSCCL have been reported. This report describes a case of PSCCL with *FGFR2* alterations in a male patient in his 50s. The patient presented with loss of appetite and epigastric pain. Computed tomography confirmed an irregular mass in the liver and lymphadenopathy in the mediastinum and right supraclavicular region. Biopsies were obtained from the liver and right supraclavicular lymph nodes, and a diagnosis of squamous cell carcinoma was determined. No other primary lesions were identified, and PSCCL was diagnosed. Administration of gemcitabine + cisplatin and gemcitabine + S-1 was discontinued due to allergic reactions. Erdafitinib was then administered; however, the disease progressed. The patient passed away 12 months after the initial treatment. No established treatment options for PSCCL are currently available. Identifying cancer-related genetic abnormalities may help in making treatment decisions.

## Introduction

Given the absence of squamous cells in the liver, most cases of squamous cell carcinoma (SCC) in hepatic tissue are metastases of malignant tumors originating from other organs [1]. Primary SCC of the liver (PSCCL) is rare, with only approximately 30 cases reported in English literature since the 1970s [2]. PSCCL is not directly derived from the liver tissue but is caused by neoplastic changes in the cyst wall and bile duct epithelium [2,3], although the pathogenesis remains unclear. Owing to its rarity, an appropriate treatment option is yet to be established, and the median survival time is reportedly 7.5 months [2,4], indicating an extremely poor prognosis. Systemic chemotherapy for PSCCL, such as 5-fluorouracil and cisplatin [5] and S-1 plus oxaliplatin [1], has been reported, but treatment has been largely ineffective.

In recent years, oncogenic panel tests have been increasingly used for drug selection, especially for rare cancers with no standard treatment options. FoundationOne® (Foundation Medicine, Inc., Cambridge, MA) is used to detect patient-specific, cancer-specific molecular structural abnormalities and guide the selection of drugs based on these abnormalities [6]. Reports on oncogenic panel testing for PSCCL that led to drug selection are scarce. We report a case of unresectable PSCCL with alteration in the gene for fibroblast growth factor receptor 2 (FGFR2), detected using FoundationOne® and treated with erdafitinib.

## Case presentation

We present the case of a 59-year-old male who presented with a three-week history of loss of appetite and epigastric pain. His past medical history included urolithiasis, diagnosed at the age of 55 years. He had a 35-pack-year smoking history (20 cigarettes daily for 35 years) and consumed 500 mL of beer daily since his 20s. Upon examination, the patient appeared well and was vitally stable. He was alert and fully oriented, with no abnormal movements or alterations in consciousness. Physical examination findings for vital signs were as follows: body temperature, 36.8 °C; blood pressure, 107/79 mmHg; pulse rate, 89 bpm; and oxygen saturation, 98% on room air. A physical assessment of the abdomen showed that it was flat and soft, with normal bowel sounds and no tenderness. No costovertebral angle (CVA) tenderness was noted upon back examination. Regarding the skin, no rashes, jaundice, or petechiae were observed. A 2-cm hard lymph node was palpated in the right supraclavicular area, raising suspicion of lymph node metastasis.

Laboratory tests revealed an elevated level of alkaline phosphatase/gamma-glutamyl transpeptidase at 536/99 IU/L (Table 1). Total bilirubin, aspartate aminotransferase, and alanine aminotransferase levels were within normal limits. A complete blood count showed leukocytosis (white blood count, 13,000/mm^3^) and thrombocytosis (platelet count, 363,000/mm^3^). Markers of viral hepatitis were negative. Elevated tumor markers were observed for carbohydrate antigen 19-9 (3,290 U/mL), carcinoembryonic antigen (28.4 ng/mL), SCC antigen (3.2 ng/mL), and alpha-fetoprotein (3.2 ng/mL).

**Table 1:** Laboratory test results of the patient on admission. CRP, C-reactive protein; AST, aspartate aminotransferase; ALT, alanine aminotransferase; ALP, alkaline phosphatase; GGT, gamma-glutamyl transpeptidase; HBsAg, hepatitis B surface antigen; CA 19-9, carbohydrate antigen 19-9; CEA, carcinoembryonic antigen; SCC, squamous cell carcinoma-related antigen

Laboratory parameter	Results	Normal values
Hemoglobin (g/dL)	15.5	16-16.5
Leucocytes (/μL)	13,000	4,000–9,000
Platelets (×10^4 ^/μL)	36.3	13.0-34.9
Urea (mg/dL)	9	8-22
Creatinine (mg/dL)	0.69	0.60-1.00
CRP (mg/dL)	5.26	<0.4
Albumin (g/dL)	3.3	3.7-4.7
AST (U/L)	26	12-30
ALT (U/L)	28	6-30
ALP (U/L)	536	195-340
GGT (U/L)	99	<50
Total bilirubin (mg/dL)	0.3	0.2-1.2
HBsAg	Negative	Negative
Hepatitis C antibody	Negative	Negative
CA 19-9 (U/mL)	3,290	<37
CEA (ng/mL)	28.4	<5.0
SCC (ng/mL)	3.2	<1.5

**Figure 1:**
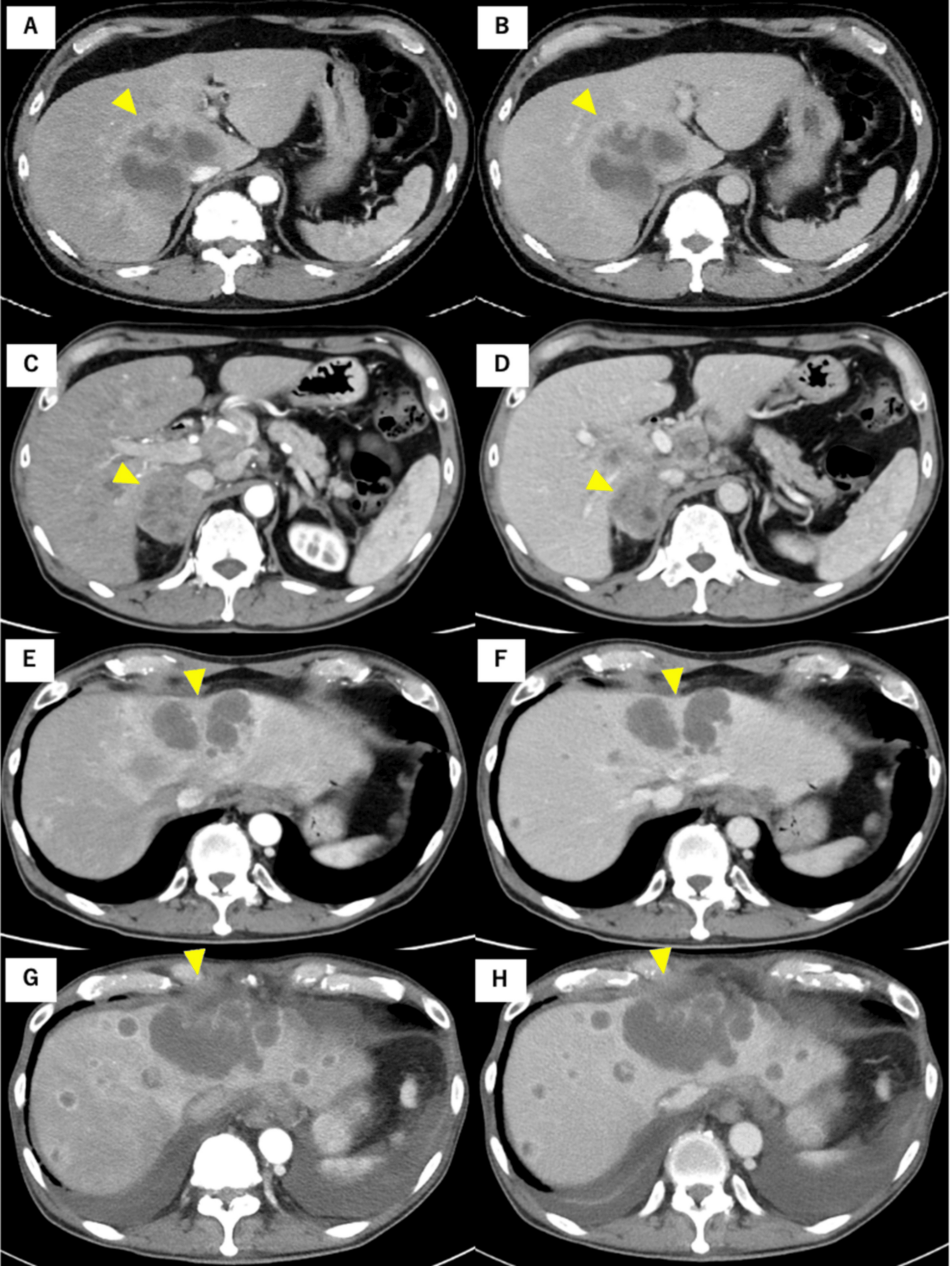
Computed tomography findings. (A) and (B) Before treatment: (A) Arterial phase and (B) portal phase showing a 77-mm low-density mass with contrast enhancement, localized to the caudate lobe and segment 7. A slight contrast effect is noted in the portal phase. (C) and (D) Six months after the first treatment: Hilar lymph nodes and right adrenal metastasis appear enlarged in both the arterial (C) and portal (D) phases. (E) and (F) Nine months after the first treatment: The intrahepatic metastasis appears enlarged in both the arterial (E) and portal (F) phases. (G) and (H) Eleven months after the first treatment: The intrahepatic metastasis has further enlarged with ascites in both the arterial (G) and portal (H) phases.

**Figure 2:**
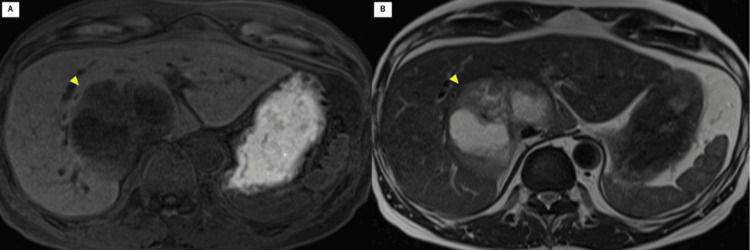
Magnetic resonance imaging. (A) T1-weighted magnetic resonance (MR) image showing a tumor with low signal intensity. (B) T2-weighted MR image showing low signal intensity at the margin and high signal intensity at the center of the tumor.

Contrast-enhanced computed tomography (CT; Figure 1 shows a time series of CT images) revealed a 77-mm low-density mass with contrast enhancement in the caudate lobe and segment 7. A slight contrast effect was noted during the portal and delayed phases (Figure 1B). Lymphadenopathy was observed in the mediastinum and right supraclavicular region. Magnetic resonance imaging (MRI) results were also evaluated (Figure 2). A low signal was detected at the tumor margin, and a high signal was detected at the tumor center on T2-weighted MRI (Figure 2B).

**Figure 3:**
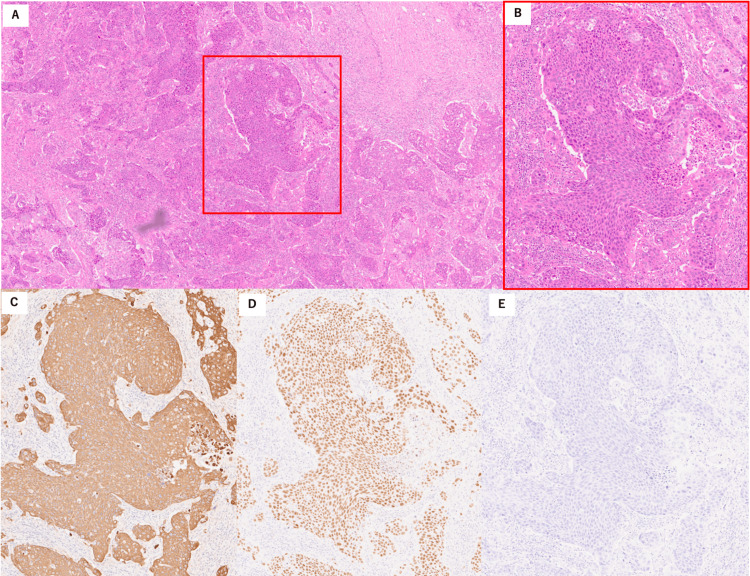
Histologic findings. (A and B) Hematoxylin and eosin staining of lymph node biopsies showing tumor cells that are nest-shaped with abnormal nuclear morphology. (C, D, and E) Immunohistology: Tumor cells are positive for cytokeratin (CK) 5/6 (C) and p63 (D), but negative for CK20 (E).

**Figure 4:**
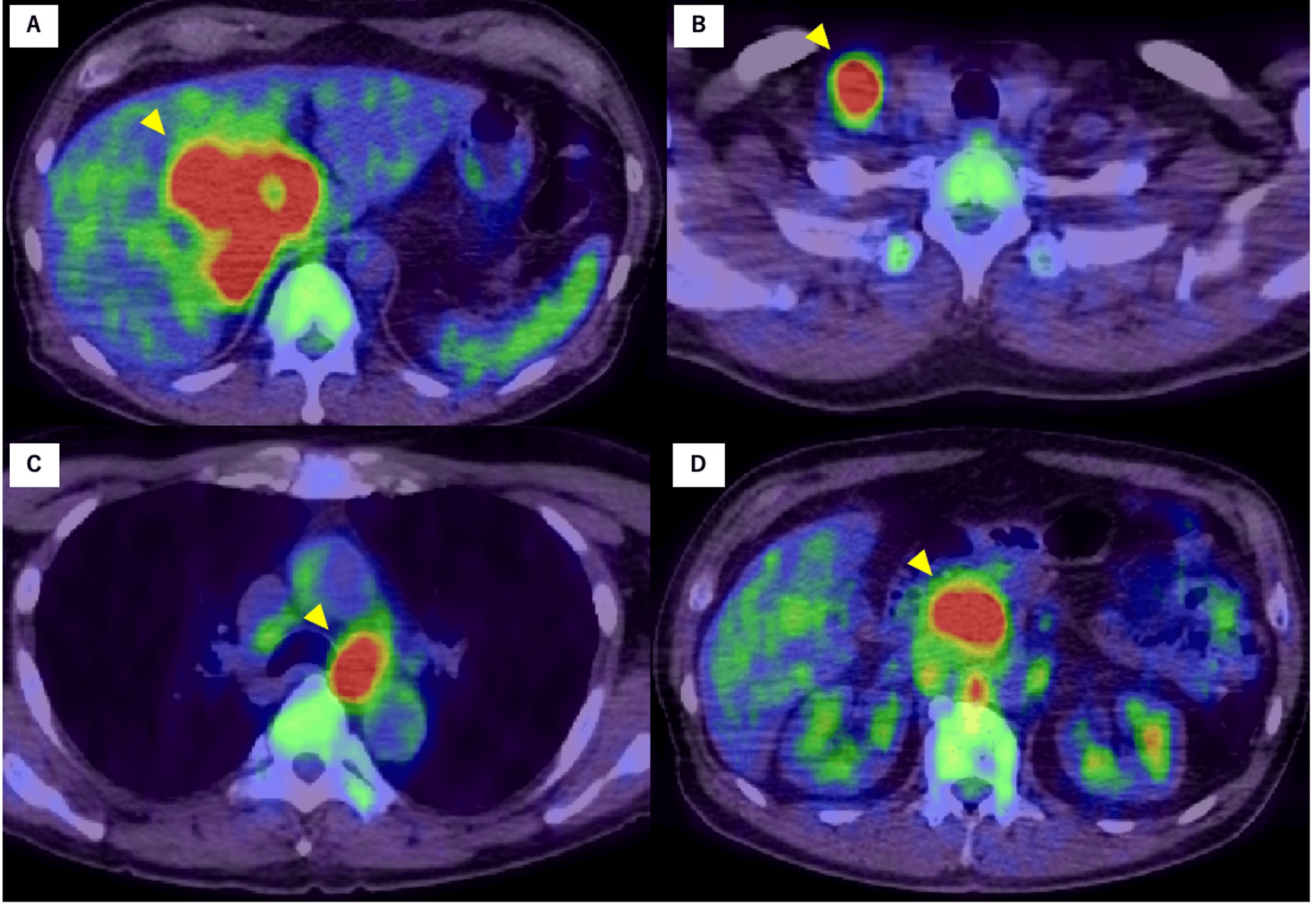
Positron emission tomography imaging. The images show increased metabolism in the (A) right liver lobe mass and (B) right supraclavicular and in (C) mediastinal and (D) para-aortic lymph nodes.

An 18F-fluorodeoxyglucose positron emission tomography/CT showed significantly increased metabolism in the right liver lobe mass, with a maximum standardized uptake value of 14.8 for 18F-fluorodeoxyglucose. Increased 18F-fluorodeoxyglucose uptake was observed in the para-aortic, right supraclavicular, and mediastinal lymph nodes, with no abnormal uptake in other regions (Figures 3A-3D). Hematoxylin and eosin staining of the right supraclavicular lymph node biopsies showed that tumor cells were nest-shaped with abnormal nuclear morphology (Figures 4A, 4B). No evident lumen formation or mucus production was observed, and differentiation into adenocarcinoma could not be confirmed. Immunohistology revealed that the tumor cells were positive for cytokeratin (CK)5/6, CK19, and p63 but negative for CK20 (Figures 4C-4E). The histopathological diagnosis was SCC. Liver tumor biopsies were also consistent with SCC, but the sample volume was insufficient. Head and neck MRI and upper and lower endoscopy were performed to identify the primary SCC, but no other primary lesions were found. The final diagnosis was PSCCL with multiple lymph node metastases.

Surgical resection was not indicated owing to the presence of distant metastases. The patient was in good general condition and had an Eastern Cooperative Oncology Group performance status of 0. Palliative chemotherapy was selected instead of surgery. A combination of gemcitabine and cisplatin (1,000 mg/m^2^ + 25 mg/m^2^, respectively) was administered but stopped immediately due to poor food intake and the development of skin rash four days after administration. FoundationOne® revealed a stable microsatellite status and a tumor mutation burden of 2.52 mut/Mb. Loss of cyclin-dependent kinase inhibitor 2A (CDKN2A) and CDKN2B and FGFR2 fusion were detected. Erdafitinib was administered two months after gemcitabine and cisplatin. Obstructive jaundice with a total bilirubin level of 10 mg/dL was detected four months after initiating erdafitinib. CT revealed enlarged hilar lymph nodes, multiple intrahepatic metastases, and right adrenal metastasis (Figures 1C, 1D). The patient was found to have a progressive disease, and erdafitinib was discontinued. He underwent endoscopic retrograde cholangiopancreatography for obstructive jaundice, and a bile duct stent was placed. Three months later, CT showed increased and growing intrahepatic metastasis (Figures 1E, 1F). Gemcitabine plus S-1 (1,000 mg/m^2^ + 80 mg/m^2^/day, respectively) was started, but the patient developed a sore throat on day 6 and facial edema on day 7 after administration. CT showed unremarkable findings. Both symptoms improved after administration of hydrocortisone. Based on the history of drug-induced skin rash after gemcitabine administration, these symptoms were considered to indicate allergy to gemcitabine. We decided to continue treatment with S-1.

The patient was hospitalized for anorexia two months after starting the S-1 therapy. CT showed an enlarged primary tumor, intrahepatic metastasis, and multiple lymph nodes, with pleural effusion and ascites (Figures 1G, 1H). As his general condition worsened, chemotherapy was discontinued, and palliative care was initiated. The patient passed away 12 months after the start of treatment.

## Discussion

PSCCL is a rare malignant disease. Both the Sixth Primary Liver Cancer Treatment Protocol and the World Health Organization classify PSCCL as a subtype of intrahepatic cholangiocarcinoma [2]. PSCCL must be distinguished from intrahepatic cholangiocarcinoma arising from adenocarcinoma of bile duct epithelial origin, metastatic liver tumors, and liver abscesses [7]. Nevertheless, distinguishing PSCCL from these diseases based on imaging alone is challenging. The definitive diagnosis should be based on pathological findings, such as liver biopsy while performing a systemic search to rule out metastatic tumors [8]. Metastatic SCC of the liver typically occurs after esophageal or gynecological cancer [9]; metastases can also originate from head and neck, skin, or lung cancer [2]. Although rare, SCC of the colon and rectum has also been reported [10-12]. In the current case, although positron emission tomography CT, whole-body CT, and upper and lower endoscopies were performed, no primary tumor capable of causing metastatic SCC of the liver was identified.

Regarding the pathological findings in PSCCL, immunohistochemical staining is usually positive for CK5/6, p63, and p40 [1]. These findings suggest a squamous epithelial origin with keratinization. In general, positive staining for CK19 suggests that the cells originate from the bile ducts [13]. A positive result for CK20 suggests gallbladder adenocarcinoma, while a negative result suggests SCC [14-16]. In our case, the tumor cells were positive for p63 and CK5/6, consistent with a diagnosis of SCC. Additionally, the cells were positive for CK19 and negative for CK20, indicating SCC of bile duct origin and a low likelihood of adenocarcinoma of the gallbladder. As previously reported [17], carcinogenesis of bile duct epithelial origin was suggested.

To date, no guidelines for treating this condition have been established. Overall survival time is longer in patients undergoing radical surgery compared to those receiving palliative treatment (median survival time of 17 vs. 5 months, *P* = 0.005, log-rank test) [2]. Surgical resection is the recommended treatment. However, PSCCL is occasionally detected at an advanced stage with distant metastases. In such cases, systemic chemotherapy, rather than surgical resection, may be an option. Based on the available literature [15], patients with PSCCL have been treated with chemotherapy, including adjuvant treatment, since 2000. Sixteen cases, including our case, are summarized in Table 2 [1,2,5,8,17-25]. Platinum-based regimens were used in 10 cases, including six cases with the 5-fluorouracil and cisplatin regimen. Historically, this has been a frequently used regimen for SCC of an unknown primary site [26,27]. Gemcitabine-based chemotherapy was initially selected for our patient because PSCCL is classified as a subtype of intrahepatic cholangiocarcinoma. Overall, although several chemotherapeutic regimens were attempted, our experience and previous reports indicate that the prognosis of patients treated with chemotherapy alone is poor.

**Table 2:** Case of primary squamous cell carcinoma of the liver treated with chemotherapy after 2000. ^*^Next-generation sequencing tests (including FoundationOne®). 5-FU, fluorouracil; CDDP, cisplatin; MTX, methotrexate; CBCDA, carboplatin; PTX, paclitaxel; L-OHP, oxaliplatin; GEM, gemcitabine; TACE, transcatheter arterial chemo embolization; LNM, lymph node metastasis

Reference	Year	Sex	Age	Tumor size/Location	Distant metastasis	Treatment	Outcome/Survival
Saito et al. [18]	2002	M	63	9 cm/left lobe	n/a	5-FU + CDDP + MTX	Dead/5 months
Hsieh et al. [19]	2005	M	65	22 cm/right lobe	None	Surgery + Radiation + 5-FU	Dead/18 months
Boscolo et al. [8]	2005	M	64	10 cm/left lobe	None	Surgery + 5-FU + CDDP	Survive/11 months
Iimuro et al. [20]	2011	F	73	10 cm/right lobe	None	Surgery + 5-FU + CDDP	Dead/13 months
Wilson et al. [21]	2013	M	34	10 cm/right lobe	None	Surgery + TACE + CBDCA + PTX	Survive/6 months
Zhang et al. [2]	2015	F	70	15 cm/right + left lobe	None	TACE + S-1 + L-OHP	Dead/9 months
Mao et al. [22]	2019	M	69	7 cm/left lobe	Peritoneum	Surgery + GEM + L-OHP	Dead/4 months
Xiao et al. [1]	2021	M	79	4 cm/right + left lobe	None	S-1 + L-OHP	Dead/20 months
Xiao et al. [1]	2021	M	47	10 cm/right lobe	None	Surgery + TACE + GEM + 5-FU	Survive/35 months
Xiao et al. [1]	2021	F	79	8 cm/right lobe	None	Anlotinib	Dead/2 months
Yamada et al. [23]	2021	M	52	8 cm/right + left lobe	Para-aortic LNM	Radiation + 5-FU + CDDP	Dead/5 months
Wang et al. [24]*	2021	M	72	11 cm/left lobe	None	Surgery + camrelizumab	Survive/14 months
Kang et al. [25]	2022	M	73	4 cm/left lobe	None	Surgery + Xindilimab	Survive/8 months
Omar et al. [5]*	2022	M	33	14 cm/right lobe	None	5-FU + CDDP	n/a
Lee et al. [17]	2022	F	61	6 cm/right lobe	None	5-FU + CDDP	Survive/8 months
Our case*	2023	M	52	7 cm/right lobe	Para-aortic, mediastinal, subclavicular LNM	GEM + CDDP, erdafitinib, pemigatinib, GEM + S-1	Dead/12 months

In recent years, FoundationOne® has played an important role in guiding the treatment of malignant tumors. However, reports on its use in PSCCL have been limited to two cases, largely owing to the rarity of this disease. In both cases, no abnormalities in cancer-related genes that required treatment were noted [5,24]. In our case, FGFR2 fusion was noted using FoundationOne®. The FGFR family of proteins plays an important role in cell proliferation and differentiation. FGFR alterations reportedly lead to carcinogenesis in various cancers [28-31]. FGFR2 fusions or rearrangements are the most common FGFR alterations in biliary tract cancers and are expressed in 15%-20% of intrahepatic cholangiocarcinomas [32]. The patient was enrolled in a clinical trial (RAGNAR, an international, single-arm, phase 2 study) [33] using erdafitinib, an FGFR tyrosine kinase inhibitor; however, treatment was discontinued because of disease progression. A recent genomic profiling analysis of cholangiocarcinoma harboring FGFR2 fusions or rearrangements showed that co-occurring CDKN2A/B alterations were associated with significantly shortened progression-free survival [34]. CDKN2A/B alterations were detected in our patient. The possibility remains that PSCCL harboring CDKN2A/B mutation is refractory to treatment. After treatment with FGFR inhibitors, a large number of secondary mutations in FGFR or stimulation of other signaling pathways have been identified in patients [35]. Secondary mutations could lead to drug resistance.

## Conclusions

PSCCL is a rare malignancy with a poor prognosis. Information regarding cancer-related genetic abnormalities in PSCCL is sparse. To our knowledge, this is the first report of PSCCL harboring an FGFR2 fusion. Erdafitinib was administered, but unfortunately, the therapeutic effect was poor. The development of appropriate treatment strategies is expected with the accumulation of additional data.
